# Challenges of implementing diagnostic‐related groups and healthcare promotion in Iran: A strategic applied research

**DOI:** 10.1002/hsr2.1115

**Published:** 2023-02-14

**Authors:** Farkhondeh Asadi, Azam Sabahi, Nahid Ramezanghorbani, Hassan Emami

**Affiliations:** ^1^ Department of Health Information Technology and Management, School of Allied Medical Sciences Shahid Beheshti University of Medical Sciences Tehran Iran; ^2^ Department of Health Information Technology, Ferdows School of Health and Allied Medical Sciences Birjand University of Medical Sciences Birjand Iran; ^3^ Department of Development & Coordination Scientific Information and Publications, Deputy of Research & Technology Ministry of Health & Medical Education Tehran Iran

**Keywords:** challenges, diagnostic related groups, DRGs, prospective payment system

## Abstract

**Background and Aim:**

Implementing the diagnostic‐related groups (DRGs) promotes the efficiency of healthcare. Therefore, the present study aimed to identify the challenges facing implementing the DRGs in Iran.

**Methods:**

The present study is a strategic applied research conducted in two phases. In the first phase, the challenges facing DRGs were extracted through a literature review. Then the collected data is entered into a checklist consisting of five sections including technological, cultural, organizational, strategic, and natural challenges. In the second phase, data were collected by purposive sampling and semistructured interviews with 10 managers of the Medical Services Organization of Tehran, Iran. Data analysis was performed by conventional content analysis using MAXQDA software and descriptive using SPSS software version 19.

**Results:**

The challenges facing the implementing DGRs from the experts' perspective included technological, organizational, nature, strategic, and cultural in order of priority. The three main fundamental challenges were reported; lack of integrating the DGRs with health information system (70%), frequent changes of management (70%), reducing the quality of care following early patient discharge (60%).

**Conclusion:**

The results of the present study showed that the DRG system faced with challenges and healthcare officials should apply policies and guidelines to reform the system before changing the reimbursement system in Iran. By considering the leading countries experiences in the nationalizing the DRG system field, the problems and solutions of the system can be identified and aid in the more successful implementation of these systems.

## 1 INTRODUCTION

The health system transformation with the aim of equitable distribution of health in society and increasing the quality of life of individuals has created the need to reform payment systems.^1^ Since diagnostic related groups (DRGs) provide a framework for monitoring the quality of treatment and the consuming services in health centers, as well as cost control, it has received particular attention in most health systems.^2^ DRGs were used to implement the prospective payment method in healthcare organizations in the 1980s and 1990s. It has also been used as a tool for allocating adequate funding, measuring the productivity of health centers, comparing the performance of centers, and even epidemiological and research studies.^3^ DRGs are a system of classifying patients based on personal characteristics, diagnoses, medical characteristics of patients, length of hospitalization, and the number of resources used and are the basis of a prospective payment system.^4^ One of the benefits of this system is helping budget planning and benchmarking health service centers, helping to improve the general financial situation, utilization management, managing and planning and reducing future expenses, optimizing organizational and operational structures, improving the performance of the repayment system, and effectiveness in hospitals, and better allocation of financial resources.^5^ In addition, it provides the possibility of predicting medical expenses and estimating the length of stay of patients.^6^, ^7^

DRGs were designed at Yale University in the late 1960s and were used as part of Medicare patient reimbursement. After reviewing the DRGs system, Medicare Severity was established that supported three distinct complication levels (substantial complication and comorbidities, complication and comorbidities, uncomplicated, and other diseases) to create clinically homogeneous groups.^8^ The main structure of DRGs was then modified by considering four levels of severity of illness, which refers to the degree of loss of function or physiologic decompensation of an organ system and risk of mortality. The All Patient‐Refined Diagnosis‐Related Groups (APR‐DRGs) classification is currently used for reimbursement in some European countries such as Belgium, Spain, Portugal and Italy, some Arab countries, and more than 30 states in the United States.^9^ Germany's DRGs are regulated under the Australian DRGs under the name G‐DRGS,^10^ although DRGs are the primary means of reimbursement hospitals. However, it may jeopardize the main goals of hospital services, including the usual motivation set by DRGs to shorten a patient's stay in certain specialties, such as intensive care, which is high risk. Also, the grouping of diagnoses related to some specialties may not be considered accurate enough, such as psychiatric care, may not accurately predict costs, or calculate the cost of some infrequently provided hospital services, such as multiple trauma care.^11^

On the other hand, the reimbursement accuracy and the beneficial relationship of the standard cost calculation plan depend on the budget's precise allocation and the calculation of a fair tariff by the hospital payment system. However, the allocation of budgets at the hospital level and the calculation of tariffs at the national level for relative hospitalization prices are rarely considered and evaluated in studies.^6^ Also, due to the time‐consuming determination of DRGs codes, preparing the relevant software should be considered in the planning related to the launch of this system.^12^ Implementation of DRGs‐based payment systems in some hospitals has been reduced, and in others, especially in the emergency department, increased.^13^ This system in Japan has significantly reduced health costs and, consequently, resources. In addition, the average patients' stay has been reduced to 2.29 days. However, as in other countries, no improvement in the quality of care has been reported.^14^, ^15^ Studies have shown that, in addition to the benefits of using DRGs in reducing overuse of health services, reducing the length of stay, and controlling treatment costs, it has led to unintended consequences such as reduced quality of care, dumping, the need for recoding, and frequent hospitalizations.^16^, ^17^

The results of Barouni's review study^18^ regarding the challenges and outcomes of the implementation of the DRG system showed that the most frequent issues were related to the increase in costs, the lack of adequate supervision and technical infrastructure, and the complexity of the method. Adverse outcomes decreased patient stay length, early patient discharge, admissions, services, and increased readmissions.

The results of Zeynep's study^19^ aimed at investigating the issues and developments in the implementation of the DRG system in France. This system has created new challenges for controlling hospital activity and ensuring the appropriateness of care provided from a medical point of view. The French DRG model should align greater efficiency with the aims of better quality and care effectiveness.

Conditions such as information systems, technical facilities, and adequate support are required to implement DRGs; there should be a document review committee to ensure compliance with relevant laws; in addition to personnel training, individuals need to be responsible for receiving relevant data, monitoring quality standards, and setting standards, and practical guidelines. During implementing the reimbursement system, while ensuring the stability of the hospital standardizing the data systems, the accuracy, comprehensiveness, and timeliness of data should be continuously monitored.^20^, ^21^

Therefore, on the one hand, considering the need to implement DRGs and their benefits, and on the other hand, the challenges of implementing this system, it is essential to be aware of the challenges before implementation in planning to reduce and eliminate it. Therefore, this study aims to investigate the challenges facing implementing DRGs in Iran.

## 2 METHODS

The present study is a strategic applied research conducted in 2020 in two phases. In the first phase the challenges facing DRGs were extracted through a literature review. For this purpose, the keywords of diagnosis‐related groups, DRG, and challenge were used in PubMed, Scopus, and Web of Science databases until September 2020. The retrieved articles were evaluated by the research team and the challenges facing the DRG based on the data extraction form were retrieved from the relevant articles.^22^, ^23^, ^24^, ^25^, ^26^, ^27^, ^28^, ^29^, ^30^, ^31^, ^32^, ^33^

Then the collected data is entered into a checklist that its reliability was confirmed by the test−retest method and *r* = 0.8 and consists of five sections including: technological, cultural, organizational, strategic, and nature challenges. The questions in each section had four options: first priority, second priority, third priority, and none. In the second phase, data were collected by purposive sampling and semistructured interviews with 10 managers, all had bachelor's (BS) or higher degrees in health information management. Inclusion criteria had at least 5 years of work experience at different levels of the Social Security and Medical Services Organization of Tehran, Iran.

The second researcher, a PhD in health information management, who had experience in an interview for qualitative studies, conducted all the interviews. She went to Social Security and Medical Services Organization, explained the study objectives to managers, invited them to participate in the study, and scheduled an interview date. All interviews were conducted face‐to‐face, audio recorded, and transcribed from January to March 2020. A 20−30 min interview was conducted. Data analysis was performed by conventional content analysis using MAXQDA, 10 software, and descriptive statistics (frequency and percentage) using SPSS software version 19.

All ethical considerations such as getting written consent, maintaining data confidentiality at all stages, the possibility of excluding at any stage, optional participation in the study, recording the interviews using the code without naming, and mutual decision about the time and place of the interview were observed.

## 3 RESULTS

### 3.1 Findings from the literature review

A total of 150 articles were retrieved. The retrieved articles were reviewed and critically evaluated by the research team. Finally, 12 full‐text English articles including the challenges of implementing diagnostic‐related groups were studied and research data were collected. The authors identified the challenges and classified them into five technological, cultural, organizational, strategic, and natural categories.

### 3.2 Results from interviews

Challenges facing the implementation of DGRs from the experts' perspective, in order of priority, included technological, organizational, nature, strategic, and cultural. Prioritizing the challenges is shown in Table 1.

**Table 1:** Percentage of prioritizing the challenges facing the implementing DGRs in Iran from the experts' perspective.

Challenges rank	Technological	Cultural	Organizational	Strategic	Nature
First	70	0	20	10	20
Second	10	10	60	10	10
Third	10	10	10	20	50
Fourth	10	10	10	50	10
Fifth	0	70	0	10	10

From the experts' perspective, the fundamental challenges of DRGs were reported in the section of technological challenges, lack of integrating the DGRs with health information system (HIS) (70%), in the section of organizational challenges, frequent changes of management (70%), in the section of nature challenges, lack of coverage of special treatments (50%), in the section of strategic challenges, lack of proper explaining the goals and capabilities of implementing DGRs (60%), and in the section of cultural challenges, decreasing the quality of care following early patient discharge (60%). Table 2 shows the classification of DRGs challenges.

**Table 2:** Distribution of frequency percentage of challenges to the implementation of DGRs from the experts' perspective.

Challenges	(Percentage)
Technological	
Lack of integrating the DGRs with HIS	(70)
Lack of a valid application to calculate the payment amount	(30)
Organizational	
Lack of support for senior management of the organization	(10)
Frequent change of management	(70)
Lack of trained personnel	(20)
Nature	
Lack of coverage of capital costs	(20)
Lack of coverage of special treatments	(50)
Lack of treatment coverage with technological innovations	(30)
Strategic	
Lack of proper explaining the goals and capabilities of implementing DGRs	(60)
Lack of legal support for the implementation of DGRs	(40)
Cultural	
Decreasing quality of care following early patient discharge	(60)
The reluctance of medical center managers to implement DRGs	(40)

Abbreviations: DRGs, diagnostic‐related groups; HIS, health information system.

## 4 DISCUSSION

The present research is a strategic applied study conducted to examine the challenges facing the implementing DRGs in Iran from the managers' perspective of different levels of the Social Security and Medical Services Organization in 2020 in Tehran. The present study results indicated that the fundamental challenges facing DRGs in Iran are in order of priority: technological, organizational, nature, strategic, and cultural.

DRGs are a method of classifying patients for health insurance purposes. In this system, patients are classified based on variables such as primary and secondary diagnosis, age, sex, complications, and treatments. Therefore, costs are controlled, and reimbursements are facilitated by third‐party providers.^22^ Although the main goals of DRGs‐based reimbursement mechanisms vary around the world, increasing transparency and efficiency, providing effective care, controlling costs, and improving the quality of care are among the essential of these goals. However, these types of systems also encounter various challenges and unintended outcomes, including that hospitals tend to accept more cost‐effective patients or pay more attention to cases that require outpatient care.

In some cases, patients are even discharged early to reduce costs.^14^, ^17^ More than half of the people mentioned a cultural challenge (decreasing the quality of care following early discharge) in the present study, consistent with the previous studies' results. It seems that policymakers and researchers should apply policies and guidelines to improve the DRGs payment system so that the quality of service delivery is not reduced while saving resources and costs.

In a study conducted by Barouni et al.^18^ to systematically examine the challenges and adverse consequences of implementing DRGs‐based reimbursement mechanisms, the challenges were presented in four categories, including:

1. Management challenges: This challenge pointed out that DRGs‐based reimbursement mechanisms are not appropriate for specific services (skincare, trauma, fibrocystic disease, heart surgery, rare diseases, urological disorders, intensive care, and care for the elderly and children). The results also indicated delays in reimbursing hospital costs, rising costs in teaching hospitals, poor maintenance of medical records, and a lack of coding rules and standards.
2. Organization environment challenges: Poor compliance guidelines, inadequate registration and possible loss of patient records, and pressure on physicians to discharge patients early.
3. Technical challenges: Includes issues with data encoding and misclassification in DRGs.
4. Personnel‐related challenges: Such as physicians' unfamiliarity with the classification of DRGs.

Their study did not address the technological challenge, while in the current study, the essential challenge facing implementing the DRGs system was the technological challenge. Given that many of the studies reviewed in Barouni's study were in the United States, the first widespread use of DRGs began in New Jersey in the late 1970s and was introduced in 1983 as part of the Medicare program in the United States^25^; therefore, it seems that the experiences of this country over the years have been able to overcome the problems facing the implementation of DRGs, including technological challenges. Hence, using the experiences of leading countries in the nationalizing DRGs in Iran to address the challenges, including technological challenges, is helpful.

Examining the status of DRGs in the United Kingdom, Australia, and Canada shows that these countries have also benefited from US experience in implementing DRGs.^34^ In general, a thorough understanding of international experiences with DRGs is essential to informing countries when developing and reviewing their national systems.^35^, ^36^ In this regard, technical and managerial capacity building at the national level is necessary to support the implementing DRGs.^31^, ^37^

In a study by Kotherova et al.,^38^ which aimed to investigate the effects of DRGs on the financing of Czech hospitals and make suggestions for possible changes in the country's hospital reimbursement mechanism, some of the essential DRGs‐related challenges identified and classified in eight categories: The need for a consistent definition of group creation, the tendency to increase the seriousness of cases, the focus on quality, the base rate convergence, repeated hospitalization and length of stay, professional coding, cost billing system, level of centralization. Also, in this study, suggestions were made to solve these problems: Considering a sufficient number of groups in DRGs, for which French, German, or Dutch examples are helpful. Applying more classification factors in the system (e.g., using patient's age as an element for group classification in some situations), considering higher weight for quality indicators in the reimbursement system, to unify rates at the national level through a regulation corridor, above a certain number of treatment days the case must be coded under a greater severity level, creating a coding manual, evaluating medical services based on the exact cost components of individual cases and taking into account highly specialized care centers (higher cost due to expensive technology, higher wages, and the like), and ultimately managing reimbursements at the national level to a lesser extent to make healthcare providers more independent.

Mihailovic stated in a study that to solve the problems facing DRGs, incentives should be created for efficient use of hospital resources by paying hospitals based on the number and type of cases treated, which guarantees a minimum level of care quality.^39^

In the United Kingdom, there are quality indicators in the DRGs reimbursement system. The UK is one of the few countries with clear rules and procedures for quality assessment. Assessing the care quality is closely related to reporting complications. Some EU countries recognize not only primary and secondary diagnoses but also comorbidities that are known at the time of hospitalization and complications from hospitalization using markers to report them.^40^ Various organizational and technical challenges related to DRGs‐based reimbursement mechanisms have been reported in some other studies, including poor records keeping systems, incorrect classification of diseases, irregularities in coding procedures and diagnoses, and a general lack of coding rules and standards.^18^, ^31^, ^41^ The present study results revealed that in the organizational dimension, frequent management change; in the nature dimension, lack of coverage of special treatments; in the technological dimension, lack of integrating the DGRs with HIS; in the cultural dimension, decreasing quality of care following early patient discharge; in the strategic dimension, lack of proper explaining the goals and capabilities of implementing DGRs, have been the fundamental challenges from the managers' perspective, which in some cases, are consistent with the previous studies' results.

However, lifestyle changes and types of patients referred to different geographical hospitals can often lead to differences in treatment patterns, medical costs, service demand, and overall disease burden, which ultimately lead to changes in the DRGs reimbursement system.^42^ On the other hand, the implementing DRGs in each country should be commensurate with each country's capacities, needs, and health conditions. However, using the experiences of leading countries in nationalizing the DRGs, identifying problems, and providing solutions in this field can help in the more successful implementation of these systems.^4^, ^36^ Although few studies have been conducted on the challenges facing the DRGs system, identifying problems in this area can help more efficient planning and policies to implement these systems better. Given the benefits of using DRGs, it is crucial to identify challenges before implementing them to plan to reduce and eliminate challenges. Essential measurements should be taken into account to localize DRGs in Iran to improve the financial system of the healthcare system.

One of the limitations of the current study was the examination of the challenges of the DRG system based on the experiences of managers of the Medical Services Organization in Tehran, and it is difficult to generalize the findings to all professional groups or managers. Therefore, to achieve more rich results, it is suggested that the problems faced by the DRG system be examined from the perspective of managers and experts in other cities.

## 5 CONCLUSION

The results of the present study showed that the DRG system faced with challenges and healthcare officials should apply policies and guidelines to reform the system before changing the reimbursement system in Iran. By considering the leading countries experiences in the nationalizing the DRG system field, the problems and solutions of the system can be identified and aid in the more successful implementation of these systems.

## AUTHOR CONTRIBUTIONS

**Farkhondeh Asadi**: Conceptualization; data curation; formal analysis; writing—review & editing. **Azam Sabahi**: Conceptualization; data curation; writing—original draft; writing—review & editing. **Nahid Ramezanghorbani**: Data curation; writing—original draft; writing—review & editing. **Hassan Emami**: Writing—original draft; writing—review & editing. All authors have read and approved the final version of the manuscript.

## CONFLICT OF INTEREST STATEMENT

The authors declare no conflict of interest.

## ETHICS STATEMENT

This study is approved under the ethical approval code of IR.SBMU.RETECH.REC.1400.520.

## TRANSPARENCY STATEMENT

The lead author Farkhondeh Asadi affirms that this manuscript is an honest, accurate, and transparent account of the study being reported; that no important aspects of the study have been omitted; and that any discrepancies from the study as planned (and, if relevant, registered) have been explained.

## Data Availability

All data generated or analyzed during this study are included in this published article. All authors have read and approved the final version of the manuscript had full access to all of the data in this study and takes complete responsibility for the integrity of the data and the accuracy of the data analysis. The data that support the findings of this study are available from the corresponding author, [Farkhondeh Asadi], upon reasonable request.
